# Malignant transformation of sinonasal inverted papilloma: A retrospective analysis of 32 cases

**DOI:** 10.3892/ol.2014.2539

**Published:** 2014-09-16

**Authors:** HUAN-XIN YU, GANG LIU

**Affiliations:** Department of Otorhinolaryngology Head and Neck Surgery, Tianjin Huanhu Hospital, Tianjin 300060, P.R. China

**Keywords:** sinonasal tumor, inverted papilloma, malignancy, squamous cell carcinoma

## Abstract

Sinonasal inverted papillomas (SNIPs) are derived from the benign tumors of the epithelial cells and have the potential to recur and exhibit malignant characteristics. The aim of the present study was to investigate the clinicopathological features and prognosis of patients with malignant transformation of SNIP. A total of 32 consecutive cases, who were patients at the Department of Otorhinolaryngology Head and Neck Surgery, Tianjin Huanhu Hospital from January 1991 to January 2008, were retrospectively reviewed. Survival rates and prognostic factors were calculated using the Kaplan-Meier method and multivariate Cox model survival analysis. The malignancy accounted for 8.99% of all types of SNIP. There were 25 males and seven females, and the median age of onset was 56.5 years. The sites of tumor included 22 in the nasal cavity and ethmoid sinuses, and 10 in the maxillary sinus. The tumors included 21 high-grade tumors, eight intermediate-grade tumors and three low-grade tumors. The number of patients with T1, T2, T3 and T4 stage disease was three, 10, 16 and three, respectively, according to the American Joint Committee on Cancer staging method. Based on the percentage of malignant cells in the entire tumor tissue, five patients had grade I tumors, five had grade II, eight had grade III and14 had grade IV. Among the 32 patients, three cases exhibited distant metastasis, and 19 patients underwent surgery plus postoperative radiotherapy, 10 underwent surgery alone and three underwent radiotherapy alone. The 5-year survival rate was 72.5% and the median overall survival time was 62.2 months. Kaplan-Meier univariate survival analysis indicated that the clinical stage and treatment method were prognostic factors, and multivariate Cox model survival analysis confirmed that the clinical stage and treatment method were independent factors for overall survival (relative risk: 4.211 and 0.312, respectively; P<0.05 for both). T3 and T4 staging and mono-treatment were associated with poor patient survival. Overall, the present study identified that the morbidity of SNIP-associated malignancy was low, the clinicopathological features were not specific, and the prognosis was improved compared with other types of sinonasal squamous cell carcinoma. The clinical stage and treatment method were found to affect the prognosis, and surgery plus postoperative radiotherapy was the predominant form of treatment. The present study may improve the understanding of the prognosis for patients with malignant SNIP in the future.

## Introduction

Sinonasal inverted papilloma (SNIP) is derived from the benign tumors of the epithelial cells and comprises 0.5–7.0% of all diagnosed sinonasal tumors. SNIP has the potential to recur and exhibit malignant characteristics, including atypia, dysplasia and and carcinoma *in situ* (1). Although the incidence of SNIP with carcinoma differs widely according to various reports (2–53%) (2), the incidence has been observed as 11% in a recent large study (2). To date, the accurate cause of SNIP is not fully understood. There are currently no reliable predictor or biological markers for recurrence or malignancy. Surgery is the main treatment option for patients with SNIP (2). The SNIP can progress to squamous cell carcinoma (SCC) with a high degree of differentiation, and has an improved outcome compared with that of pure primary SCC following treatment (3,4). SCC often occurs in the SNIP itself, presenting as atypical hyperplasia or cancer of various histological stages (5). The present study retrospectively analyzed the clinical data of 32 cases of malignant SNIP. The associations between the clinical/histopathological characteristics of the patients and SNIP malignancy, along with their association with patient survival, were analyzed in order to improve the understanding of the prognosis of patients with malignant SNIP.

## Patients and methods

### Clinical data

A total of 356 SNIP patients received treatment at the Department of Otorhinolaryngology Head and Neck Surgery, Tianjin Huanhu Hospital (Tianjin, China) between January 1991 and January 2008. Of these, 32 cases were pathologically confirmed to have malignant SNIP. Therefore, the present study retrospectively analyzed the data of the 32 cases, which included patient age, gender, disease location, clinical staging and surgical treatment. The study was approved by the ethics committee of Tianjin Huanhu Hospital and written informed consent was obtained from all patients.

### Histopathological analysis

All pathological sections were analyzed by three highly qualified pathologists who were blinded to patient data. According to the differentiation of malignant cells, tumor sections were classified into three differentiation levels, well-, moderately and poorly differentiated according the the classification by the World Health Organization (6). In a typical tissue section, the percentage of malignant cells was graded as follows: I, ≤25% malignant cells; II, 26–50% malignant cells; III, 51–75% malignant cells; IV, ≥76% malignant cells (6).

### Follow-up procedure

All patients were followed up, for periods ranging between 23 to 212 months, with an interval of three months between each follow-up, by telephone and letter. The patients’ referral statuses were obtained, so as to evaluate the current condition and tumors status of the patients.

### Statistical analysis

SPSS 17.0 software (SPSS, Inc., Chicago, IL, USA) was used for statistical analysis. Kaplan-Meier survival curves and the log-rank test were utilized to analyze the survival rate of patients, while the Cox regression model was used for the multivariate analysis. P<0.05 was considered to indicate a statistically significant difference.

## Results

### Clinical characteristics

**Figure 1:**
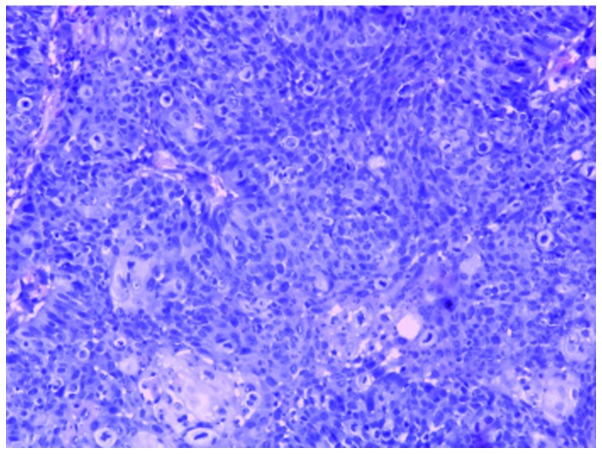
Representative image of the progression from sinonasal inverted papilloma to squamous cell carcinoma. The papillary epithelial cells exhibit a disordered arrangement, the basement membrane disappears, the nuclei are deeply stained and become larger, chromatin thickening with atypia is evident and pathological nuclear fission is visible (hematoxylin and eosin staining; magnification, ×100).

**Figure 2:**
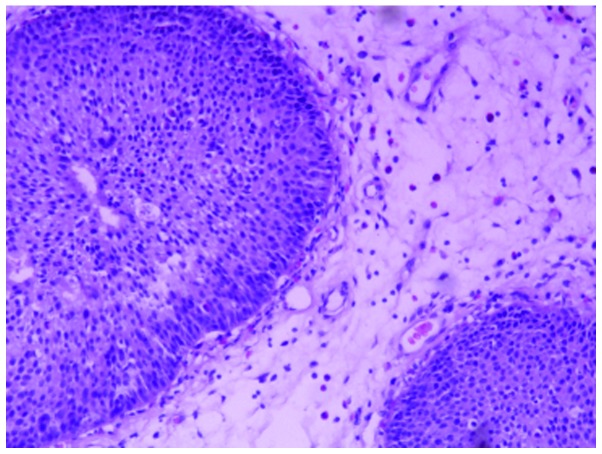
Representative image of sinonasal inverted papilloma. The section shows papillary epithelial cells undergoing the process of endogenous growth. The cells are arranged in neat rows and the basement membrane is very integrated (hematoxylin and eosin staining; magnification, ×100).

**Figure 3:**
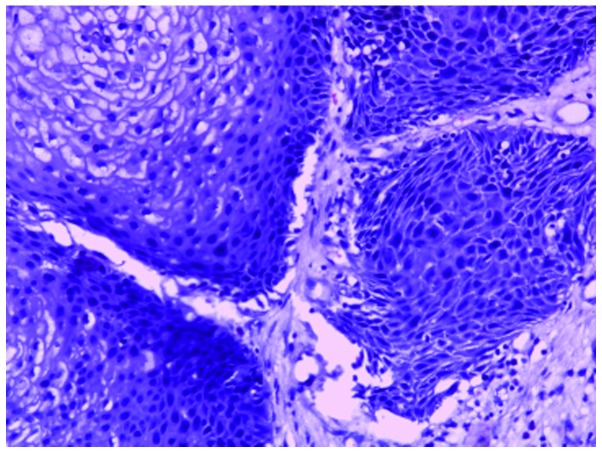
Representative image of sinonasal inverted papilloma with atypical hyperplasia. The section shows the transitional cell growth of papillary epithelial cells. The cells have a disordered arrangement and the nuclei are of different sizes. Atypic epithelial cells and severe dysplasia are evident (hematoxylin and eosin staining; magnification, ×100).

In the same period of continuous treatment of 356 patients with SNIP, 8.99% (32/356) of patients exhibited the malignant type. These 32 patients were selected for the retrospective analysis and comprised 25 males and seven females, with a male to female ratio of 3.6:1. The median age of onset was 56.5 years old. All 32 patients were diagnosed with pathologically malignant SNIP that had progressed to SCC (Fig. 1). Nasal inverted papilloma was the diagnosis in 18 patients, which included two cases of pathological nasal inverted papilloma dysplasia (Fig. 2), and the remaining 16 cases were of pathological nasal inverted papilloma with atypical hyperplasia (Fig. 3). In the present study, the corresponding incidence rates were 3.9% (14/356), and 5.1% (18/356), respectively.

The site in which the tumor occurred was the maxillary sinus in 10 cases, and the nasal cavity and sinus in 22 cases. The site had extended to the nasal sinuses or outside in 19 cases, which included 10 cases in the orbit, seven cases in the skull and two cases in the pterygopalatine fossa. According to the American Joint Committee on Cancer staging method (7), there were three cases of T1, 10 of T2, 16 of T3 and three of T4 (Table I). Three patients had distant metastases, including two cases of metastasis to the brain and one case of metastasis to the lung.

Among the 32 patients, 10 received surgery alone, three received radiotherapy alone and 19 underwent both surgery and radiotherapy (Table I). Surgical treatment included endoscopic resection in eight cases, combined endoscopic nasal surgery in 13 cases and simple nasal surgery in eight cases.

For all patients, the follow-up period ranged from 23 to 212 months, and the median survival time was 74.8 months. From the follow-up, it was concluded that of the 20 patients who did not survive, nine cases were of uncontrolled local tumor (time between end of treatment and recurrence, ≤3 months), eight cases were of local tumor recurrence (time between end of treatment and recurrence, >3 months) and three cases involved distant metastasis.

### Histological features

Among the 32 cases, 21 were well-differentiated, eight were moderately differentiated and three were poorly differentiated. According to the percentage of malignant areas throughout the entire tumor tissue, tumor tissue sections were divided into four stages: Five cases of grade I, five of grade II, eight of grade III and 14 of grade IV (Table I).

### Analysis of survival rate and prognostic factors

**Figure 4:**
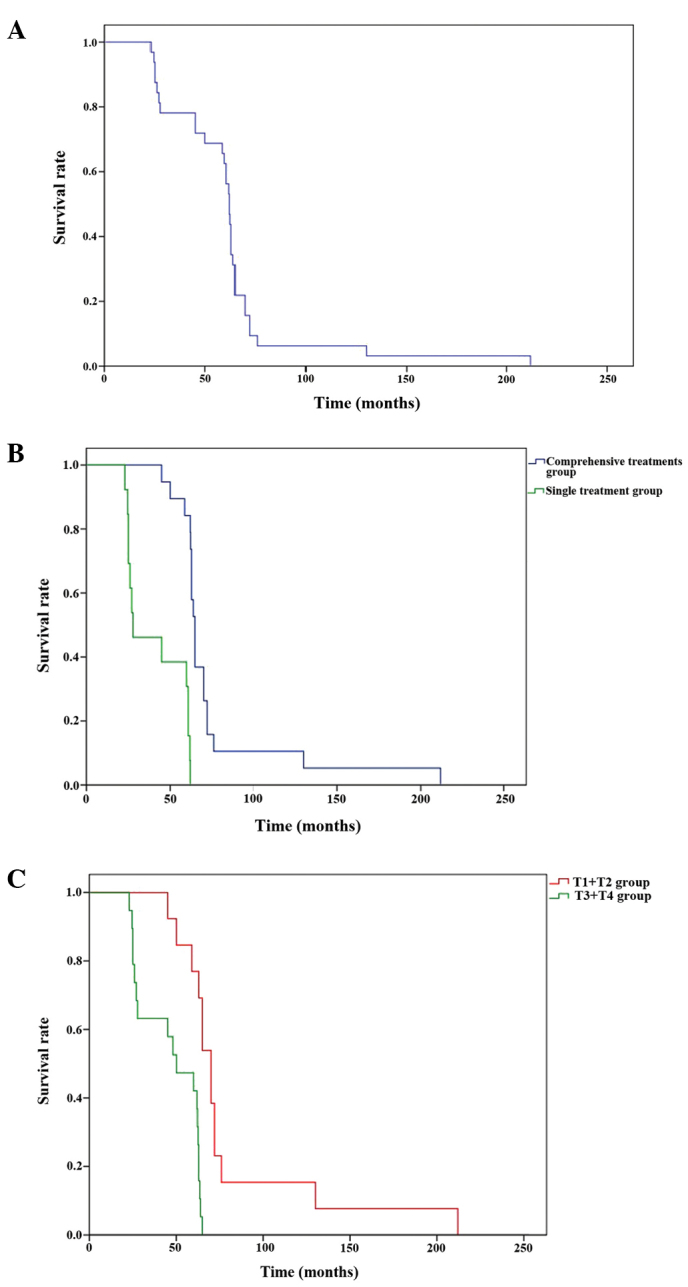
Kaplan-Meier curves. (A) Kaplan-Meier curve for overall survival rate. (B) Kaplan-Meier curve for survival rate according to treatment method. (C) Kaplan-Meier curve for survival rate according to clinical stage.

The Kaplan-Meier method was used to calculate the 5-year survival rate of patients, which was determined to be 72.5%. The median survival time was 62.2 months (Fig. 4A). The 5-year survival rate and median survival time of patients who received comprehensive treatment (both surgery and radiotherapy) were 83.8% and 64.2 months, respectively. However, the 5-year survival rate and median survival time of patients who received a single treatment (either surgery or radiotherapy) were 40.7% and 27.7 months (Fig. 4B). The difference in 5-year survival rate and the median survival time between the two treatment groups was statistically significant (P=0.006 and P=0.005, respectively).

The effect of the following factors on survival was then analyzed: Gender, age, clinical stage, disease site, orbital violation, skull base violation, surgical treatment, the degree of tumor cell differentiation and the proportion of malignant cells. According to the Kaplan-Meier method analysis and the log-rank test, the factors affecting patient survival were the clinical stage (P=0.002), orbital violation (P=0.005), skull base violation (P=0.009) and treatment method (P=0.006) (Table I). Additionally stages T3 and T4 (Fig. 4C), orbital violation, skull base violation and monotherapy were associated with decreased patient survival rates. However, gender, age, disease site, the degree of tumor cell differentiation and the proportion of malignant cells did not affect patient survival (Table I). Multivariate Cox regression analysis showed that clinical stage (P=0.004) and treatment method (P=0.032) were independent risk factors for survival (Table II).

## Discussion

Currently, the specific mechamisms underlying SNIP malignancy remain unclear. From the perspective of pathogenesis, SNIPs are borderline tumors, and they can undergo malignant transformation with disease progression. Most commonly, SNIP malignancy is associated with SCC, followed by malignant adenocarcinoma, while small cell carcinoma is rarely seen in the clinic (8). SCC that originates from the papilloma often has a high level of differentiation, and the prognosis of which is improved compared with simple primary SCC. Even in the late stages of the disease, lymph node metastases are rare (3,4). All pathological types within the 32 patients of malignant transformation of SNIP were SCC, well differentiated SCC accounted for 65.6% (21/32) of the malignant cases. Zhangzong *et al* (9) analyzed the clinical data of 146 cases of primary SCC of the nasal sinus and the overall 5-year survival rate was 49.1%. In the present study, the overall survival rate of the 32 patients with malignant SNIP was 72.5%. Therefore, from this data, the prognosis of patients with malignant SNIP appears to be improved compared with that of patients with primary SCC.

There are two main ways in which SNIPs may become malignant: The SNIP and malignancy may occur in the same lesion, or the malignancy may occur in the site from which an SNIP was previously resected (6). Mirza *et al* (10) followed up 65 patients for 20 years and reported that the incidence rate of SCC occurring simultaneously with inverted papilloma was 7.1%, while the incidence rate of SCC occurring independently of the papilloma was 3.6%. In the present study, the corresponding incidence rates were 3.9% (14/356), and 5.1% (18/356), respectively.

In this study, the incidence of malignancy among SNIP cases was 8.99% SNIP (32/356), and this was similar to that reported in the literature, which reported that the incidence of malignancy among SNIP cases was 11% (3,11). The number of males with malignant SNIP was greater than the corresponding number of females (3.6:1), and the median age of onset for malignant cases was 56.5 years. There was no significant difference in the 5-year survival rate between patients with early (≤60 years) and late (>60 years) onset of disease. In the present study cohort, gender and age were not significant factors affecting the prognosis of patients with malignant SNIP (P=0.285 and P=0.567, respectively).

Sinus computed tomography scan and brain magnetic resonance imaging can show the range of tumor tissue types preoperatively, and may aid in the clinical classification of the tumor and the corresponding preoperative preparation. Preoperative biopsy is an ineffective method of identifying malignant SNIP and SNIPs mostly become malignant from the center; therefore, multiple preoperative biopsies should be obtained from different locations in the tumor tissue, particularly near the base of the central area of tumor, so as to prevent misdiagnosis (2,3). In the current study, 27 out of the 32 patients were diagnosed with concomitant SNIP and SCC, or SCC *in situ* by preoperative biopsy. The remaining five cases were diagnosed with SNIP or severe dysplasia, pathologically confirmed as SCC, by preoperative biopsy.

The results of this study showed that the main factors affecting the prognosis of patients with malignant SNIP were clinical stage and treatment method. Due to the malignant SNIP, such patients often have a long history of illness and repeated surgery, and the tumor invades an extensive range. As the sinonasal cavity is proximal to the important organizational base of the skull, en bloc resection of the tumor is difficult to perform safely, and chemotherapy should be administered for local control following surgery (12). In the present study of 32 patients, orbit and skull base invasion of the tumor were identified in 10 and seven cases, and the 5-year survival rates were 50.8 and 45.5% (64.2 vs. 27.7 months; P=0.006), respectively, suggesting a poor prognosis.

With the development of endoscopic surgical techniques for the treatment of malignant SNIP, the traditional method of open surgical procedures has been replaced by minimally invasive endoscopic surgery or endoscopic-assisted surgery combined with chemotherapy, improving survival rates (?). Surgery plus postoperative radiotherapy and chemotherapy has been found to be the most effective treatment (6,13). In the present study, the 19 patients who received surgery plus postoperative radiotherapy had a significantly improved 5-year survival rate (83.8%) compared with that of the remaining 13 patients who received monotherapy (either surgery or radiotherapy) (83.8 vs 40.7%; P=0.006). The median survival time was also significantly improved in the patients receiving comprehensive treatment compared with that in the patients receiving monotherapy (64.2 vs. 27.7 months; P=0.006). We conclude that complete removal of the tumor, to ensure negative margins and facilitate thorough histological examination, supplemented with postoperative radiotherapy, can improve the prognosis of malignant SNP.

SNIP has a low incidence of malignancy, a lack of specific clinical manifestations and biological indicators; currently, identification of SNIP mainly relies on the pathological diagnosis. In the present study, clinical stage and treatment modality were independent risk factors for prognosis. We propose that the primary treatment modality for malignant SNIP should comprise surgical excision with postoperative radiotherapy and chemotherapy. In further studies, a comphrensive analysis of patient clinical stage, treatment and other risk factors must be performed to improve patient survival rates.

## Figures and Tables

**Table I:** Log-rank single-factor analysis for survival patients.

Factor	Number of cases	5-year survival rate (%)	*χ*^2^ value	P-value
Gender			1.875	0.285
Male	25	71.3		
Female	7	73.3		
Age of onset (years)			0.237	0.567
≤60	15	74.2		
>60	17	62.8		
Diseased part			0.365	0.512
Nasal cavity and sinus	22	67.2		
Maxillary sinus	10	73.0		
Pathological grade			0.056	0.728
Poor and moderately differentiated	11	34.4		
Well differentiated	21	65.6		
Clinical stage			9.782	0.002
T1+T2	13	78.7		
T3+T4	19	47.9		
Orbital violation			8.765	0.005
None	22	72.8		
All	10	50.8		
Skull base violation			5.231	0.009
None	25	70.1		
All	7	45.5		
Treatment method			7.565	0.006
Comprehensive treatment	19	83.8		
Monotherapy	13	40.7		
Malignancy grade			0.436	0.486
I+II	10	73.2		
III+IV	22	70.1		

**Table II:** Cox regression multivariate analysis.

Factor	Partial regression coefficient	Standard error	Wald *χ*^2^	DOF	P-value	RR	95% CI
Gender	1.231	0.236	0.030	1	0.924	0.924	0.942–8.901
Age	1.632	0.430	0.121	1	0.760	1.211	0.672–7.099
Diseased part	1.152	0.260	2.451	1	0.248	0.912	0.661–6.351
Pathological grade	0.532	0.277	1.454	1	0.432	0.852	0.872–4.554
Clinical stage	1.452	0.512	8.400	1	0.004	4.211	1.512–11.321
Orbital violation	1.532	0.470	2.701	1	0.105	3.321	1.472–7.211
Skull base violation	0.866	0.596	2.876	1	0.096	3.433	1.855–6.462
Treatment method	−1.182	0.526	4.387	1	0.032	0.312	0.121–0.922
Malignancy grade	1.455	1.198	1.697	1	0.291	1.122	0.635–6.251

DOF, degrees of freedom; RR, relative risk; 95% CI, 95% credibility interval.
